# Holographic is Hamiltonian, relatively

**DOI:** 10.1007/s11005-026-02137-w

**Published:** 2026-08-27

**Authors:** Piotr T. Chruściel, Raphaela Wutte

**Affiliations:** 1 Beijing Institute of Mathematical Sciences and Applications, Huairou, China; 2 Center for Theoretical Physics of the Polish Academy of Sciences, Warsaw, Poland; 3 Mathematical Sciences and STAG Research Centre, University of Southampton, Highfield, Southampton, SO17 1BJ UK

**Keywords:** Energy, Holographic energy, Asymptotically Anti-de Sitter spacetimes, 83C40

## Abstract

We show that a relative holographic energy coincides with the relative Hamiltonian energy.

## Introduction

Consider a stationary (n+1)-dimensional spacetime , with ${\overline{g}}$ solving the vacuum Einstein equations with a negative cosmological constant. (Large families of such metrics exist by [1, 7, 8, 21].) Suppose that  admits a conformal boundary at infinity  *à la Penrose* with a, say smooth, conformal class of metrics induced on . Each such metric ${\overline{g}}$ comes with a family of vacuum, dynamical, metrics  which share their conformal class at infinity with ${\overline{g}}$ [16, 19]. For each such metric *g* one can define both its holographic energy [23] (see also [2, 4, 14, 17, 25]) and its Hamiltonian energy relative to ${\overline{g}}$ [5]. The aim of this note is to show that a holographic energy of *g* defined relatively to ${\overline{g}}$ coincides with the Hamiltonian energy.

This fact has previously been pointed out in spacetime dimensions 3+1 in [10], but the result here holds in all dimensions n+1≥4 and for any conformal metric on . (The case n+1=3 requires separate treatment, cf. e.g. [11] and references therein.)

In this article we are concerned with the Hamiltonian energy as defined in [5], based on the formalism developed in [20]. The approach is related to, but does not coincide with, a subset of the much more extensive treatment in [23] where the holographic charges are considered from the perspective of the covariant phase space analysis in the spirit of [22, 26].

In retrospect, our calculations here give a tacit way of implementing the counterterm-subtraction of [23].

As such, we have assumed a stationary background, as the Hamiltonian charge associated with a vector field *X* which is timelike at large distances and is a Killing vector field for the metric ${\overline{g}}$ usually has the interpretation of energy. One can similarly define relative Hamiltonian charges for any ${\overline{g}}$-Killing vector *X*, regardless of its causal character, concluding again that for vacuum metrics *g* and ${\overline{g}}$ which share the same conformal structure at  the Hamiltonian charge of *g* relative to ${\overline{g}}$ equals the difference of holographic charges of *g* and ${\overline{g}}$; see Section 5 for more comments on this.

## Holographic charges

When the matter fields decay sufficiently fast and the spacetime admits a smooth conformal completion at infinity, there exist coordinates in which the metric can be written in the form

2.1 $$\begin{aligned} g&= x^{-2} \big ( dx^2 + ({\mathring{\gamma }}_{AB} + x^2 {{\mathop {\gamma }\limits ^{(2)}}}_{AB} + \ldots + x^n \log x {{\mathop {\gamma }\limits ^{(\textrm{log})}}}_{AB} + x^n {{\mathop {\gamma }\limits ^{(n)}}}_{AB} + \ldots ) dy^A dy^B \big ) \end{aligned}$$

2.2 $$\begin{aligned}&=: x^{-2} (dx^2 + \gamma _{AB} dy^A dy^B) \,, \end{aligned}$$

where for n≥3 [14]

2.3 $$\begin{aligned} {{\mathop {\gamma }\limits ^{(2)}}}_{AB} = - \frac{1}{n-2} ({\mathring{R}}_{AB} - \frac{1}{2(n-1)} {\mathring{R}} {\mathring{\gamma }}_{AB})\,. \end{aligned}$$

Recall some results reviewed or derived in [15] for Einstein metrics *g*. Keeping in mind that *n* is the dimension of the boundary, if *n* is odd then

$$ {{\mathop {\gamma }\limits ^{(n)}}}:= {{\mathop {\gamma }\limits ^{(n)}}}_{AB} dx^A dx^B $$

is ${\mathring{\gamma }}$-trace-free and ${\mathring{\gamma }}$-divergence-free. If *n* is even, then the trace and divergence of ${{\mathop {\gamma }\limits ^{(n)}}}$ are determined locally in terms of the boundary metric, and are nonzero in general. This is contained in [15, Theorem 4.8]. The divergence is identified explicitly when n=4, and [15, Equation (3.19)] identifies the trace explicitly for n=4,6. It is a consequence of the results in [15, Chapter 7] that the trace and divergence vanish whatever *n* if the boundary metric is locally conformally Einstein, in particular if it is locally conformally flat.

Set

2.4 $$\begin{aligned} {\tau }\equiv {\tau }_{AB}dx^Adx^B := \big ( {{\mathop {\gamma }\limits ^{(n)}}}_{AB}- \frac{1}{n} {\mathring{\gamma }}^{CD} {{\mathop {\gamma }\limits ^{(n)}}}_{CD} {\mathring{\gamma }}_{AB}\big ) \big |_{x=0} dx^A dx^B \,. \end{aligned}$$

Given a conformal Killing vector *X* of the conformal structure of  and a section ${\mathcal {C}}$ of , when the divergence of τ vanishes (in particular when *n* is odd, or when the metric is asymptotically locally BK — see Subsection 3.1 — whatever *n*, or when the boundary metric is locally conformally Einstein whatever *n*), it is natural to define *holographic-type charges* as

2.5 $$\begin{aligned} {q}[{\mathcal {C}},X](g) = - \int _{{\mathcal {C}}} {\mathring{\gamma }}^{AC}{\tau }_{CB}X^B dS_A \,, \end{aligned}$$

where $dS_A$ is given by $\sqrt{-\det {\mathring{\gamma }}}\, \partial _A\, \rfloor dx^1\wedge \cdots \wedge dx^n$. Then q is the same for all sections of . We emphasise that our calculations below show that q arises naturally from a Hamiltonian analysis of the theory regardless of the vanishing of the divergence of τ, for any vector field *X* for which the Hamiltonian volume integrals converge; compare Section 5.

Recall that the *holographic energy momentum tensor* t is defined as [25, Equation (17)]

2.6 $$\begin{aligned} {t}_{AB} = \frac{n }{16 \pi G} ({{\mathop {\gamma }\limits ^{(n)}}}_{AB} + X^{(n)}_{AB}) \,, \end{aligned}$$

with $X^{(n)}_{AB} = 0$ for odd *n*. The field $X^{(n)}_{AB}$ depends only upon the fields ${\mathring{\gamma }}$ and ${{\mathop {\gamma }\limits ^{(a)}}}$ with 1≤a<n and their derivatives, it vanishes in odd space dimensions and its exact form depends on the spacetime dimension as explained in the paragraph following Equation (1.3) of [14]^1^. Hence, upon requiring that the matter fields decay to sufficiently high order at , it is sufficient to know ${\mathring{\gamma }}$ and ${{\mathop {\gamma }\limits ^{(n)}}}$ to calculate ${t}_{AB}$.

The charges Q are then defined as [4, Equation (3.17)] (compare [13, 14])

2.7 $$\begin{aligned} {Q}[{\mathcal {C}},X](g) = - \int _{{\mathcal {C}}} {\mathring{\gamma }}^{AC}{t}_{CB}X^B dS_A \,. \end{aligned}$$

Hence Q differs from q by a multiplicative factor and, for even *n*, a shift depending only upon the metric ${\mathring{\gamma }}$ and its derivatives. According to [4, Equation (3.16)] the tensor field ${t}_{AB}$ is divergence free in any dimension.

Alternatively, the energy-momentum tensor can also be expressed as [24, Equation (49)] (see also [4, Equation (3.15)])

2.8 $$\begin{aligned} {t}_{AB} = - \frac{1}{8 \pi G} \left( K_{(n) AB} - ({\mathring{\gamma }}^{CD} K_{(n) CD}) {\mathring{\gamma }}_{AB}\right) \,, \end{aligned}$$

where $K_{(n)}$ are certain expansion coefficients of the extrinsic curvature of constant *x* slices in terms of eigenfunctions of the so-called dilation operator.

While the divergence of (2.4) does not vanish generically, this tensor is convenient in the analysis below due to its simple form. The corresponding charge (2.5) differs from (2.7) (aside from a multiplicative factor) only by a term which cancels in the relative charge $Q[{\mathcal {C}},X](g) - Q[{\mathcal {C}},X](\bar{g})$ between two metrics *g* and $\bar{g}$ sharing the same conformal infinity.

## Hamiltonian charges

Given a vector field *X* and a spacelike hypersurface  with boundary $\partial {\mathcal {S}}$ included in , consider the Hamiltonian charges as derived in [5]

3.1 $$\begin{aligned} H[{\mathcal {S}},X,{\overline{g}}](g) = \int _{\partial {{\mathcal {S}}}} E^{{\mu }{\nu }} \eta _{{\mu }{\nu }} \,, \end{aligned}$$

with ${\mu },{\nu }\in \{0,\ldots ,n\}$, where ${\overline{g}}$ is a background metric with respect to which the mass will be defined. Further $\eta _{{\mu }{\nu }} = \partial _{\mu }\rfloor \partial _{\nu }\rfloor (d t \wedge dx^1 \wedge ... \wedge dx^{n-1} \wedge dx)$ and

3.2 $$\begin{aligned} E^{{\mu }{\nu }} = E^{{\mu }{\nu }}_K - E^{{\mu }{\nu }}_K \vert _{g = {\overline{g}}}+ \frac{1}{8\pi } X^{[{\mu }} Z^{{\nu }]} \sqrt{|\det {\overline{g}}|} \,, \end{aligned}$$

with

3.3 $$\begin{aligned} E^{{\mu }{\nu }}_K = \frac{\sqrt{|\det g|}}{16 \pi }(\nabla ^{\mu }X^{\nu }- \nabla ^{\nu }X^{\mu }) \,, \end{aligned}$$

3.4 $$\begin{aligned} Z^{\mu }= e(g^{{\mu }{\nu }} C^{\rho }_{{\nu }{\rho }} - g^{{\nu }{\rho }} C_{{\nu }{\rho }}^{\mu }) \,, \qquad C^{\mu }_{{\nu }{\rho }} = \Gamma ^{\mu }_{{\nu }{\rho }} - {\bar{\Gamma }}^{\mu }_{{\nu }{\rho }} \,, \end{aligned}$$

and $e = \sqrt{\det ({\overline{g}}^{{\mu }{\rho }} g_{{\rho }{\nu }})}$, where $\bar{\Gamma }^{\mu }_{{\nu }{\rho }}$ denotes Christoffel symbols of the metric $\bar{g}$.

### Asymptotically Birmingham-Kottler metrics

In this section we consider (n+1)-dimensional Lorentzian metrics *g* which asymptote to the background metric ${\overline{g}}$ given by

3.5 $$\begin{aligned} {\overline{g}}= {-} \left( \beta +\frac{r^2}{\ell ^2}\right) dt^2 + \frac{dr^2}{\left( \beta +\frac{r^2}{\ell ^2}\right) } + r^2 {{\mathring{\gamma }}}\,, \quad {{\mathring{\gamma }}}= {{\mathring{\gamma }}}_{{a}{b}}(x^C)dy^a dy^b \,, \end{aligned}$$

with $\beta \in \{0,\pm 1\}$, where $(x^{\mu })\equiv (t,y^a,r)$, and where $\ell ^2$ is a constant inversely proportional to the cosmological constant.

The ${\overline{g}}$-Killing vector *X* is taken to be $\partial _t$, and the spacelike hypersurface is ${\mathcal {S}}=\{t={\text {const}}\}$.

We use the following ${\overline{g}}$–orthonormal frame:

3.6 $$\begin{aligned} f  _{{\hat{0}}}= \frac{1}{\sqrt{\beta + \frac{r^2}{\ell ^2}}}\partial _t\,, \quad f  _{{\hat{n}}}= {\sqrt{\beta + \frac{r^2}{\ell ^2}}} \partial _r\,, \quad f  _{{\hat{a}}} = \frac{1}{r} {\varphi }_{{\hat{a}}}\,, \end{aligned}$$

where ${\varphi }_{{\hat{a}}}$ is an orthonormal (ON) frame for the metric ${{\mathring{\gamma }}}$. Here, and in what follows in the current section, we use

$$ {a}\in \{1,...,n-1\} \,, $$

and we shall use hatted indices to denote the components of a tensor field in the frame $f  _{{{\hat{{\mu }}}}}$ defined in (3.6).

Let the tensor field ${e}^{{\mu }{\nu }}$ be defined by the formula

3.7 $$\begin{aligned} {e}^{{\mu }{\nu }} := g^{{\mu }{\nu }}-{\overline{g}}^{{\mu }{\nu }} \,, \end{aligned}$$

thus

$$ {e}^{{\mu }{\nu }}\partial _{\mu }\otimes \partial _{\nu }= {e}^{{{\hat{{\mu }}}}{{\hat{{\nu }}}}}f  _{{{\hat{{\mu }}}}}\otimes f  _{{{\hat{{\nu }}}}} \,. $$

We will say that a metric is *asymptotically Birmingham-Kottler* if there exists ϵ>0 such that in the frame (3.6) it holds

3.8 $$\begin{aligned} {e}^{{\hat{{\mu }}}{\hat{{\nu }}}}= {O(}r^{-n/2-\epsilon })\,, \quad f  _{{\hat{{\rho }}}}({e}^{{\hat{{\mu }}}{\hat{{\nu }}}})= {O(}r^{-n/2-\epsilon }) \,, \quad \det ( {\overline{g}}^{{\hat{{\mu }}}{\hat{{\nu }}}} + {e}^{{\hat{{\mu }}}{\hat{{\nu }}}}) - 1 = {O(}r^{-n-\epsilon })\,. \end{aligned}$$

For matter fields with finite integrated energy, this guarantees convergence of the Hamiltonian mass and is optimal when pointwise-decay conditions are imposed.

A calculation leads to the following simple expression for the *Hamiltonian mass* ${H[{\mathcal {S}},\partial _t,{\overline{g}}](g)}$ of asymptotically Birmingham-Kottler metrics:

3.9 $$\begin{aligned} {H[{\mathcal {S}},\partial _t,{\overline{g}}](g)}= &   \lim _{R\rightarrow \infty } \frac{R^n}{16\pi } \int _{{\mathcal {S}}\cap \{r=R\}} \left[ \left( \frac{1}{\ell ^2}+ \frac{\beta }{r^2} \right) \left( {\frac{r \partial \big ({\overline{g}}_{{{\hat{a}}}{{\hat{b}}}}{e}^{{{\hat{a}}}{{\hat{b}}}} \big )}{\partial r}} - (n-1) {e}^{{\hat{n}}{\hat{n}}} \right) \right. \nonumber \\  &   \left. + \frac{\beta }{ {r^2} } {\overline{g}}_{{{\hat{a}}}{{\hat{b}}}}{e}^{{{\hat{a}}}{{\hat{b}}}} \right] d^{n-1}\mu _{{{{\mathring{\gamma }}}}} \,. \end{aligned}$$

In spacetime dimension n+1=4 this simplifies to the expression given in [10]:

3.10 $$\begin{aligned} {H[{\mathcal {S}},\partial _t,{\overline{g}}](g)}= &   \lim _{R\rightarrow \infty } \frac{R^3}{16\pi \ell ^2} \int _{{\mathcal {S}}\cap \{r=R\}} \left[ r \frac{ \partial {e}^{{{\hat{1}}}{{\hat{1}}}} }{\partial r} + r \frac{ \partial {e}^{{{\hat{2}}}{{\hat{2}}}} }{\partial r} - 2 {e}^{{\hat{3}}{\hat{3}}} \right] d^{n-1}\mu _{{{{\mathring{\gamma }}}}} \,. \end{aligned}$$

If in addition to (3.8) we assume that

3.11 $$\begin{aligned} {e}^{{\hat{{\mu }}}{\hat{{\nu }}}}= {O(}r^{-n} )\,, \quad f  _{{\hat{{\rho }}}}({e}^{{\hat{{\mu }}}{\hat{{\nu }}}})= {O(}r^{-n}) \,, \end{aligned}$$

which is the fall-off rate for Birmingham metrics, Equation (3.9) can be rewritten in a form similar to (3.10) in higher dimensions as well:

3.12 $$\begin{aligned} {H[{\mathcal {S}},\partial _t,{\overline{g}}](g)}= &   \lim _{R\rightarrow \infty } \frac{R^n}{16\pi \ell ^2} \int _{{\mathcal {S}}\cap \{r=R\}} \left[ {r {\overline{g}}_{{{\hat{a}}}{{\hat{b}}}}\frac{\partial {e}^{{{\hat{a}}}{{\hat{b}}}} }{\partial r}} - (n-1) {e}^{{\hat{n}}{\hat{n}}} \right] d^{n-1}\mu _{{{{\mathring{\gamma }}}}} \,. \end{aligned}$$

### General smooth 

Consider (n+1)-dimensional metrics of the form

3.13 $$\begin{aligned} g_{{\mu }{\nu }}(x,x^A) = {\overline{g}}_{{\mu }{\nu }}(x,x^A) +x^{n-2} h_{{\mu }{\nu }}(x,x^A) \,, \end{aligned}$$

with ${\mu }, {\nu }\in \{0,\ldots ,n\}$, where ${\overline{g}}$ is a background metric with respect to which the mass will be defined. We use a Fefferman-Graham coordinate system

$$(x^{\mu }) \equiv ( y^A,x)\equiv (t,x^1,\ldots ,x^{n-1},x) \equiv (x^0,x^1,\ldots ,x^{n-1},x) \,, $$

thus

$$ g_{xx} = {\overline{g}}_{xx} = x^{-2},\quad g_{xA} = {\overline{g}}_{xA} = 0, $$

and

3.14 $$\begin{aligned} g^{{\mu }{\nu }} = {\overline{g}}^{{\mu }{\nu }} - x^{n-2} h^{{\mu }{\nu }} + ... \,, \end{aligned}$$

with the indices on $h_{{\mu }{\nu }}$ raised and lowered with ${\overline{g}}_{{\mu }{\nu }}$. (Note that in the asymptotically BK case the leading order terms of the field $e^{\mu \nu }$ of (3.7) coincide with the leading order terms of the field $-x^{n-2}h^{\mu \nu }$.)

Let us assume that both metrics *g* and ${\overline{g}}$ are vacuum. Since *g* and ${\overline{g}}$ share the same smooth conformal infinity, their asymptotic expansions at $\{x=0\}$ to the order made explicit in (3.13) will coincide, including logarithmic terms if any. It follows that the coordinate components $h_{{\mu }{\nu }}$ of the tensor field $h_{{\mu }{\nu }}dx^{\mu }dx^{\nu }$ defined in (3.13) satisfy $h_{x {\mu }}\equiv 0$ and

3.15 $$\begin{aligned} h_{AB} = \mathring{h}_{AB} + O(x\ln x) \,, \ \mathring{h}_{{\mu }{\nu }}:= h_{{\mu }{\nu }}|_{x=0} \,, \ \partial _x h_{AB} = O(\ln x) \,, \ \partial _A h_{BC} = O(1) \,. \end{aligned}$$

We note that much weaker asymptotic conditions would suffice for finite, well defined Hamiltonian charges. However, the formulae below would need a careful reexamination. This is irrelevant in the current context in any case, as we want to determine the Hamiltonian charges for metrics which have a well defined holographic mass.

We also note that matter fields which decay sufficiently fast will not change the calculations here.

Denoting by $\mathring{D}$ the covariant derivative operator of the metric $\mathring{\gamma }_{AB}$ of (2.1), one finds

3.16 $$\begin{aligned} g^{tA} C^{\rho }_{A {\rho }}&= \frac{x^{n-2}}{2} g^{tA} {\overline{g}}^{BC}\overline{\nabla }_A h_{BC} +O(x^{2n+2}) \nonumber \\&= \frac{x^{n+2}}{2} {\mathring{\gamma }}^{tA} {\mathring{\gamma }}^{BC}\overline{\nabla }_A h_{BC} \big (1+ O(x^2) \big ) +O(x^{2n+2}) \nonumber \\&= \frac{x^{n+2}}{2} {\mathring{\gamma }}^{tA} {\mathring{\gamma }}^{BC}\mathring{D}_A {\mathring{h}}_{BC} +O(x^{n+3}) = O(x^{n+2}) \,,\end{aligned}$$

3.17 $$\begin{aligned} g^{{\nu }{\rho }} C^t_{{\nu }{\rho }}&= x^{n +2} \gamma ^{AB}{\overline{\gamma }}^{tC} \left( \overline{\nabla }_A h_{BC} - \frac{1}{2} \overline{\nabla }_C h_{AB} \right) +O(x^{2n+2}) \nonumber \\&= x^{n +2} {\mathring{\gamma }}^{AB}{\mathring{\gamma }}^{tC} \left( \mathring{D}_A {\mathring{h}}_{BC} - \frac{1}{2} \mathring{D}_C {\mathring{h}}_{AB} \right) \big (1+ O(x)\big ) +O(x^{2n+2}) \nonumber \\&= x^{n +2} {\mathring{\gamma }}^{AB}{\mathring{\gamma }}^{tC} \left( \mathring{D}_A {\mathring{h}}_{BC} - \frac{1}{2} \mathring{D}_C {\mathring{h}}_{AB} \right) +O(x^{n+3}) = O(x^{n+2}) \,. \end{aligned}$$

Since $e = 1 + O(x^{{n}})$, we obtain

3.18 $$\begin{aligned} Z^t = x^{n +2} {\mathring{\gamma }}^{AB}{\mathring{\gamma }}^{tC} \left( \mathring{D}_C {\mathring{h}}_{AB} - \mathring{D}_A {\mathring{h}}_{BC} \right) +O(x^{n+3}) = O(x^{n+2}) \,. \end{aligned}$$

Next,

3.19 $$\begin{aligned} g^{xx} C^{\rho }_{x {\rho }}&= \frac{ {n}}{2} x^{n-1} {\overline{g}}^{AB} h_{AB} + O(x^{n+2}) = \frac{n}{2} x^{n+1} {\mathring{\gamma }}^{AB} {\mathring{h}}_{AB} + O(x^{n+2}) \,, \end{aligned}$$

3.20 $$\begin{aligned} g^{{\nu }{\rho }} C^x_{{\nu }{\rho }}&= - \frac{(n-2)}{2} x^{n-1} {\overline{g}}^{AB} h_{AB} + O(x^{{n+2}}) \nonumber \\&= - \frac{(n-2)}{2} x^{n+1} {\mathring{\gamma }}^{AB} {\mathring{h}}_{AB} + O(x^{{n+2}}) \,, \end{aligned}$$

so that

3.21 $$\begin{aligned} Z^x = {(n-1)} x^{n+1} {\mathring{\gamma }}^{AB} {\mathring{h}}_{AB} + O(x^{{n+2}}) \,. \end{aligned}$$

Furthermore,

3.22 $$\begin{aligned} E^{xt}_K&= \frac{\sqrt{|\det g|}}{16 \pi }(\nabla ^x X^t - \nabla ^t X^x) \nonumber \\  &= \frac{\sqrt{|\det g|}}{16 \pi }g^{x {\mu }} g^{t {\nu }} (\partial _{\mu }X_{\nu }- \partial _{\nu }X_{\mu }) \nonumber \\  &= \frac{\sqrt{|\det g|}}{16 \pi }g^{x x} g^{t {\nu }} (\partial _x X_{\nu }- \partial _{\nu }X_x) \nonumber \\&= \frac{\sqrt{|\det g|}}{16 \pi } g^{tA} \left( x^2 \partial _x(g_{AB} X^B) - \partial _A X^x \right) \,. \end{aligned}$$

To continue, we assume that *X* is a Killing vector of ${\overline{g}}$, possibly up to error terms which decay fast:

3.23 $$\begin{aligned} \overline{\nabla }_{\mu }X_{\nu }+ \overline{\nabla }_{\nu }X_{\mu }= o(x^{{s}-2}) \,, \end{aligned}$$

where $o(x^{{s}-2})$ is meant in terms of tensor components in Fefferman-Graham coordinates. The reader should think of the exponent s as the one needed for finiteness of the integral in (3.1), with possibly *o* replaced by *O*, but in the current calculations we do not make any assumptions about s until explicitly indicated otherwise.

For conformally smooth backgrounds the vector field *X* necessarily extends smoothly to the conformal boundary at infinity , with the extension tangent to the conformal boundary, its restriction to the boundary being a conformal Killing vector of the conformal metric on . One can without loss of generality assume that the coordinates $(x,y^A)$ are simultaneously Fefferman-Graham coordinates both for the background ${\overline{g}}$ and the metric of interest *g*. Writing

3.24 $$\begin{aligned} {\overline{g}}= x^{-2} (dx^2 + {\overline{\gamma }}_{AB} dy^A dy^B) \,, \end{aligned}$$

where

$$ {\overline{\gamma }}_{AB}(x,y^C) = {\mathring{\gamma }}_{AB}(y^C) + O(x^2) \,, $$

the asymptotic Killing equations in Fefferman-Graham coordinates

3.25 $$\begin{aligned} X^{\mu }\partial _{\mu }{\overline{g}}_{{\nu }{\rho }} + \partial _{\nu }X^{\mu }{\overline{g}}_{{\mu }{\rho }} + \partial _{\rho }X^{\mu }{\overline{g}}_{{\mu }{\nu }} = o(x^{{s}-2}) \end{aligned}$$

with $${\nu }{\rho }=xx$$ read

3.26 $$\begin{aligned} -2 x^{-3} X^x = \&- 2 x^{-2}\partial _x X^x + o(x^{{s}-2}) \,. \end{aligned}$$

Integrating in *x* we conclude that, for ${s}\ne 0$ (the case ${s}=0$ would need separate and irrelevant attention, as it would introduce annoying log terms),

3.27 $$\begin{aligned} X^x = \&x {\mathring{X}}^x + o(x^{{s}+1}) \,, \ \partial _x {\mathring{X}}^x=0 \,. \end{aligned}$$

Inserting this into (3.25) with ${\nu }{\rho }=xA$ gives

3.28 $$\begin{aligned} X^A = {\mathring{X}}^A - {\frac{x^2}{2} } \mathring{\gamma }^{AC} \partial _C {\mathring{X}}^x \big (1 + O(x^{2}) \big ) + o(x^{{s}+1}) \,, \ \partial _x {\mathring{X}}^A=0 \,, \ {s}\not \in \{-1,0\} \,, \end{aligned}$$

where the new restriction on s arises again to avoid dealing with further terms involving logx.

Returning to (3.22), for s>0 we can write

3.29 $$\begin{aligned} E^{xt}_K |_{g={\overline{g}}} = \  &\frac{\sqrt{|\det {\overline{g}}|}}{16 \pi } {\overline{g}}^{tA} \left( x^2 \partial _x({\overline{g}}_{AB} X^B) - \partial _A X^x \right) \,, \nonumber \\ E^{xt}_K = \  &\frac{\sqrt{|\det g|}}{16 \pi } g^{tA} \left( x^2 \partial _x(g_{AB} X^B) - \partial _A X^x \right) \nonumber \\ = \  &E^{xt}_K |_{g={\overline{g}}} + \frac{\sqrt{|\det {\mathring{\gamma }}|}}{16 \pi } \Big [ \frac{x}{2} {\mathring{\gamma }}^{CD}{\mathring{h}}_{CD} {\mathring{\gamma }}^{tA} \left( x^2 \partial _x(x^{-2} {\mathring{\gamma }}_{AB} {\mathring{X}}^B) - {x} \partial _A {\mathring{X}}^x \right) \nonumber \\&- x\, {\mathring{\gamma }}^{tC}{\mathring{\gamma }}^{AD}{\mathring{h}}_{CD} \left( x^2 \partial _x(x^{-2} {\mathring{\gamma }}_{AB} {\mathring{X}}^B) - {x} \partial _A {\mathring{X}}^x \right) \nonumber \\&+ x^{-n+1} {\mathring{\gamma }}^{tA} \left( x^2 \partial _x(x^{n-2} h_{AB} {\mathring{X}}^B) \right) \Big ] + o(1) \nonumber \\ = \  &E^{xt}_K |_{g={\overline{g}}} + \frac{\sqrt{|\det {\mathring{\gamma }}|}}{16 \pi } \Big [ \big (- {\mathring{\gamma }}^{CD} {\mathring{\gamma }}^{tA} + {2} {\mathring{\gamma }}^{tC}{\mathring{\gamma }}^{AD} \big ) {\mathring{h}}_{CD} {\mathring{\gamma }}_{AB} {\mathring{X}}^B \nonumber \\&+ (n-2) {\mathring{\gamma }}^{tA} {\mathring{h}}_{AB} {\mathring{X}}^B \Big ] + o(1) \,. \end{aligned}$$

Hence

3.30 $$\begin{aligned} E^{xt}_K - E^{xt}_K |_{g={\overline{g}}} = \  &\frac{\sqrt{|\det {\mathring{\gamma }}|}}{16 \pi } \Big [ - {\mathring{\gamma }}^{CD} {\mathring{h}}_{CD} {\mathring{\gamma }}_{AB} + n {\mathring{h}}_{AB} \Big ] {\mathring{\gamma }}^{tA} {\mathring{X}}^B + o(1) \,. \end{aligned}$$

When

3.31 $$\begin{aligned} \partial {\mathcal {S}}=\{t=0\}\cap \{x=0\} \text{ and } {s}>0 \end{aligned}$$

we obtain

3.32 $$\begin{aligned} H = \&- \int _{\partial {{\mathcal {S}}}} E^{xt} \, {d^{n-1} y} \nonumber \\ = \  &\frac{1}{16 \pi } \int _{\partial {\mathcal {S}}} \Big ( \big [ {\mathring{\gamma }}^{CD} {\mathring{h}}_{CD} {\mathring{\gamma }}_{AB} - n {\mathring{h}}_{AB} \big ] {\mathring{\gamma }}^{tA} {\mathring{X}}^B \nonumber \\&+ {(n-1)} {\mathring{X}}^t{\mathring{\gamma }}^{AB} {\mathring{h}}_{AB} \Big ) \sqrt{|\det {\mathring{\gamma }}|} {d^{n-1} y} \nonumber \\ = \  &\frac{n}{16 \pi } \int _{\partial {\mathcal {S}}} \Big ( {\mathring{\gamma }}^{CD} {\mathring{h}}_{CD} {\mathring{\gamma }}_{AB} - {\mathring{h}}_{AB} \Big ) ( \mathring{D}^{A}t ){\mathring{X}}^B \sqrt{|\det {\mathring{\gamma }}|} {d^{n-1} y} \,, \end{aligned}$$

where the overall minus sign arises from the fact that the *outer* pointing normal to the level sets of *x* is $-x\partial _x$.

### Examples

As an example, consider the case where ${\mathring{X}}^{\mu }\partial _{\mu }= {\mathring{X}}^t \partial _t$, with *H* usually interpreted as the total energy if moreover ${\mathring{X}}^t=1$. One obtains

3.33 $$\begin{aligned} H = \&\frac{1}{16 \pi } \int _{\partial {\mathcal {S}}} \Big ( - n {\mathring{h}}_{At} \mathring{D}^A t + {n} {\mathring{\gamma }}^{AB} {\mathring{h}}_{AB} \Big ){\mathring{X}}^t \sqrt{|\det {\mathring{\gamma }}|} {d^{n-1} y} \,. \end{aligned}$$

As a consistency check, let us apply (3.12) to metrics of the form (3.14). For definiteness assume that ℓ=1, and for *r* large define *x* through the equation

3.34 $$\begin{aligned} \frac{dx}{x} = - \frac{dr}{\sqrt{\beta +r^2 }} \,. \end{aligned}$$

We choose the solution with the asymptotics

3.35 $$\begin{aligned} x = \frac{1}{r}- \frac{\beta }{4r^3} +O(r^{-5}) \,. \end{aligned}$$

Then $e^{{\hat{{\mu }}}{\hat{{\nu }}}} = - x^n h^{{\hat{{\mu }}}{\hat{{\nu }}}} + O(x^{n+2})$ with $h^{{\hat{n}}{\hat{n}}} =0$, $r\partial _r =-\big (x +O(x^3)\big )\partial _x$, and (3.12) with x=1/r becomes, with $(f_{\hat{A}}) \equiv (f_{\hat{0}},f_{{\hat{a}}})$,

3.36 $$\begin{aligned} {H[{\mathcal {S}},\partial _t,{\overline{g}}](g)}= &   \lim _{x\rightarrow 0} \frac{n}{16\pi } \int _{{\mathcal {S}}\cap \{r=R\}} {\overline{g}}_{{{\hat{a}}}{{\hat{b}}}} h^{{{\hat{a}}}{{\hat{b}}}} d^{n-1}\mu _{{{{\mathring{\gamma }}}}} \nonumber \\= &   \lim _{x\rightarrow 0} \frac{n}{16\pi } \int _{{\mathcal {S}}\cap \{r=R\}} \big ( {\overline{g}}_{\hat{A} \hat{B} } h^{\hat{A} \hat{B}} + h_{\hat{0} \hat{0}} \big ) d^{n-1}\mu _{{{{\mathring{\gamma }}}}} \,. \end{aligned}$$

The volume condition in (3.8), namely

3.37 $$\begin{aligned} \det (e^{{\hat{{\mu }}}{\hat{{\nu }}}}) - 1 = {O(}r^{-n-\epsilon }) \,, \end{aligned}$$

requires

3.38 $$\begin{aligned} {\overline{g}}_{\hat{A} \hat{B} } h^{\hat{A} \hat{B}}|_{x=0} \equiv {\mathring{\gamma }}^{CD} {\mathring{h}}_{CD} =0 \,. \end{aligned}$$

When ${\overline{g}}$ is the Birmingham-Kottler metric (3.5) with ℓ=1, and if $X=\partial _t$ we recover (3.32).

As another example, let *g* be a perturbation of a Siklos wave ${\overline{g}}$, where

3.39 $$\begin{aligned} {\overline{g}}= x^{-2} \big ( -2 du ds + f(s,x,{y^a}) ds^2 + dx^2 +(dy^1)^2 + \ldots + (dy^{n-2})^2 \big ) \,, \end{aligned}$$

with $$a\in \{1,\ldots ,n-2\}$$ and

3.40 $$\begin{aligned} f(s,x,{y^a}) = {\mathring{f}}(s, y^a) + x^2 {{\mathop {f}\limits ^{(2)}}}(s,x,{y^a}), \end{aligned}$$

where both ${\mathring{f}}$ and ${{\mathop {f}\limits ^{(2)}}}$ are smooth on the conformally completed manifold. The metric ${\overline{g}}$ satisfies the vacuum Einstein equations with negative cosmological constant Λ=-n(n-1)/2 if and only if the null energy density $\bar{\rho }$ vanishes, where

3.41 $$\begin{aligned} \bar{\rho }:= \  &-\frac{1}{2} \Big (\big (\partial _{y^1}\big )^2 + \ldots + \big (\partial _{y^{n-2}}\big )^2 + \partial _x^2 - \frac{n-1}{x} \partial _x \Big ) f \nonumber \\ =\  &\frac{1}{x^2} \left( x \partial _x f - \frac{1}{2} \Delta _{\mathbb {H}^{n-1}} f\right) \,, \end{aligned}$$

where we used the Laplacian $\Delta _{\mathbb {H}^{n-1}}$ of the (n-1)-dimensional hyperbolic metric

3.42 $$\begin{aligned} x^{-2}\big (dx^2 + (dy^1)^2 + \ldots {+} (dy^{n-2})^2 \big ) \,. \end{aligned}$$

Indeed, in n+1 space-time dimensions we have (cf. [18, p.22])

3.43 $$\begin{aligned} G_{{ss}} - \frac{n (n-1)}{2} g_{{ss} }= \bar{\rho }\,. \end{aligned}$$

with the remaining components of the energy-momentum tensor vanishing.

As an example, consider the equation $\bar{\rho }=0$ in (3+1)-dimensions with $f(s,x,{y}) = \Re (\mathfrak {f}(x) e^{i{l} y})$, ${l} \in \mathbb {Z}$. We then have

3.44 $$\begin{aligned} \bar{\rho }\equiv \frac{{l}^{2}}{2} \mathfrak {f}(x) + \frac{1}{x}\frac{d \mathfrak {f}(x)}{dx} - \frac{1}{2}\frac{d^{2} \mathfrak {f}(x)}{dx^{2}} = 0 \,. \end{aligned}$$

The solutions to this equation are

3.45 $$\begin{aligned} \mathfrak {f}(x)= \frac{c_1 (\sinh ({l} x)-{l} x \cosh ({l} x))}{{l}} + \frac{c_2 ({l} x \sinh ({l} x)-\cosh ({l} x))}{{l}} \,. \end{aligned}$$

It follows that general vacuum Siklos waves can be built as finite or infinite sums of such solutions. It would be interesting to analyse whether or not any of the resulting spacetimes have good global properties.

In (3+1)-dimensions the boundary metric $\lim _{x\rightarrow 0} x^2 {\overline{g}}|_{dx=0}$ has vanishing Ricci scalar, with the only non-vanishing component of its Ricci tensor $\mathring{R}_{AB}$ being ${\mathring{R}}_{ss}=- \frac{1}{2} \partial _y^2 {\mathring{f}}$, and the only non-zero component of its Cotton tensor $\mathring{C}_{AB}$ being $\mathring{C}_{ss}= \frac{1}{2} \partial _y^3 {\mathring{f}}$. Hence a three-dimensional boundary metric is conformally flat if and only if $\partial _y^3 {\mathring{f}}\equiv 0$.

We have $$\det {\mathring{\gamma }}= - 1$$,

$$ {\mathring{\gamma }}^{tA} = \mathring{D}^A t = {\mathring{\gamma }}^{BA}\frac{\partial t}{\partial x^B } = - \frac{\partial t}{\partial s } \delta ^A_u - {\mathring{f}}\frac{\partial t}{\partial u } \delta ^A_u - \frac{\partial t}{\partial u } \delta ^A_s + {\sum _{a=1}^{n-2}} \frac{\partial t}{\partial y^{{a} }} \delta ^A_{y^{{a}}} \,, $$

and $X = \partial _u$ so that ${\mathring{X}}^t= \partial t / \partial u$. For this background (3.32) reads

3.46 $$\begin{aligned} H = \frac{n}{16\pi } \int _{\partial {\mathcal {S}}} \left( - {\mathring{h}}_{Au}\mathring{D}^{A} t + {\mathring{\gamma }}^{AB} {\mathring{h}}_{AB} \frac{\partial t}{\partial u} \right) ds \, d^{n-2} y \,, \end{aligned}$$

where now the coordinates on  are $(u,s,y^a)$, and $\partial {\mathcal {S}}$ is defined implicitly by the equation $t(u,s,y^a)=0$. When $\mathring{f} >0$ a possible choice, compatible with the spacelike character of ${\mathcal {S}}$, is $\partial {\mathcal {S}}=\{u=0\}$, i.e. .

## Equality

To conclude, let

4.1 $$\begin{aligned} g&= x^{-2} \big ( dx^2 + ({\mathring{\gamma }}_{AB} + x^2 {{\mathop {\gamma }\limits ^{(2)}}}_{AB} + \ldots + x^n \log x {{\mathop {\gamma }\limits ^{(\textrm{log})}}}_{AB} + x^n {{\mathop {\gamma }\limits ^{(n)}}}_{AB} + \ldots ) dy^A dy^B \big ) \end{aligned}$$

4.2 $$\begin{aligned} {\overline{g}}&= x^{-2} \big ( dx^2 + ({\mathring{\gamma }}_{AB} + x^2 \overline{{{\mathop {\gamma }\limits ^{(2)}}}}_{AB} + \ldots + x^n \log x \overline{{{\mathop {\gamma }\limits ^{(\textrm{log})}}}}_{AB} + x^n \overline{{{\mathop {\gamma }\limits ^{(n)}}}}_{AB} + \ldots ) dy^A dy^B \big ) \nonumber \\  &= x^{-2} \big ( dx^2 + ({\mathring{\gamma }}_{AB} + x^2 {{\mathop {\gamma }\limits ^{(2)}}}_{AB} + \ldots + x^n \log x {{\mathop {\gamma }\limits ^{(\textrm{log})}}}_{AB} + x^n \overline{{{\mathop {\gamma }\limits ^{(n)}}}}_{AB} + \ldots ) dy^A dy^B \big ) \,. \end{aligned}$$

Assuming that the traces of ${{\mathop {\gamma }\limits ^{(n)}}}_{AB}$ and $\overline{{{\mathop {\gamma }\limits ^{(n)}}}}_{AB}$ coincide (which, as already pointed out, is the case for the metrics under consideration when the matter fields decay sufficiently fast), we have

4.3 $$\begin{aligned} {\mathring{h}}_{AB} = {{\mathop {\gamma }\limits ^{(n)}}}_{AB}-\overline{{{\mathop {\gamma }\limits ^{(n)}}}}_{AB} \,. \end{aligned}$$

Hence4.44.54.6as claimed. Thus, either (2.5) or (2.7) arises from a Hamiltonian analysis, as the difference between these charges drops out of the relative Hamiltonian energy.

## Which vectors *X* and metrics ${\overline{g}}$?

Some comments about the conditions set so far are in order.

In the Hamiltonian calculations of [6] it has been assumed that the vector field *X* is a Killing vector of ${\overline{g}}$. This condition was useful to analyse the convergence of the integrals. Moreover, solutions such as Minkowski spacetime or Anti-de Sitter spacetime provide natural asymptotic backgrounds for many metrics of interest, and therefore the condition seemed to be natural in the problem at hand at the time.

So let us consider the question of convergence of the integrals appearing in this work. It follows from the calculations above that *the equalities* (4.4)-(4.6) *hold for any pair of vacuum metrics* *g* *and* ${\overline{g}}$ *which share the same conformal metric at* , *and for all vectors* *X* *extending smoothly to the conformal boundary of the spacetime, with the right-hand side of* (4.6) *being finite for such vectors*. This takes care of the question of the convergence of the integral defining *H*, and shows that neither ${\overline{g}}$-Killing vectors in spacetime, nor conformal Killing vectors of , are needed for (4.6).

However, a definition is only useful if it carries useful information. This is the case when *X* is a conformal Killing vector and when one of the tensors ${\tau }_{AB}$ or ${t}_{AB}$ is divergence-free and trace-free, for then the associated charge integrals are independent of the choice of a section of . In such a case a metric ${\overline{g}}$ for which *X* is a Killing vector which is asymptotically timelike, whenever there is one, provides a natural reference value of energy. As an example, one can prove positivity of *H* for suitable classes of asymptotically Birmingham-Kottler metrics [9, 27], and for asymptotically Horowitz-Myers metrics [3], and for small perturbations of static ${\overline{g}}$’s which themselves are small perturbations of the Anti-de Sitter metric [12]. Whether or not the charges q, or Q, or *H*, provide useful information in more general situations remains to be seen.

## Data Availability

No data were created or analyzed in this manuscript.

## Footnotes

1 In principle, *h* enters in the formula for the energy-momentum as well, but the authors have a holographic argument to remove it, see [14, text under (3.13)].

## References

[1] Anderson, M.T., Chruściel, P.T., Delay, E.: Non-trivial, static, geodesically complete spacetimes with a negative cosmological constant. II. , AdS/CFT correspondence: Einstein metrics and their conformal boundaries, IRMA Lect. Math. Theor. Phys., vol. 8, Eur. Math. Soc., Zürich, arXiv:gr-qc/0401081, 165–204 (2005)

[2] Balasubramanian, V., Kraus, P.: A Stress tensor for Anti-de Sitter gravity. Commun. Math. Phys. **208**, 413–428 (1999). arXiv:hep-th/9902121

[3] Brendle, S., Hung, P.K.: Systolic inequalities and the Horowitz-Myers conjecture, (2024). arXiv:2406.04283 math.DG

[4] Cheng, M.C.N., Skenderis, K.: Positivity of energy for asymptotically locally AdS spacetimes. JHEP **08**, 107 (2005). arXiv:hep-th/0506123

[5] Chruściel, P.T.: On the Relation Between the Einstein and the Komar Expressions for the Energy of the Gravitational Field. Ann. Inst. H. Poincaré Phys. Theor. **42**, 267 (1985)

[6] Chruściel, P.T.: Asymptotic estimates in weighted Hölder spaces for a class of elliptic scale-covariant second order operators. Ann. Fac. Sci. Toulouse Math. (5). **11**, 21–37 (1990)

[7] Chruściel, P.T., Delay, E.: Non-singular, vacuum, stationary spacetimes with a negative cosmological constant. Ann. Henri Poincaré **8**, 219–239 (2007)

[8] Chruściel, P.T., Delay, E., Klinger, P.: Non-singular spacetimes with a negative cosmological constant: IV. Stationary black hole solutions with matter fields, Class. Quantum Grav. **35**(3), 035007 (2018). arXiv:1708.04947 [gr-qc]

[9] Chruściel, P.T., Herzlich, M.: The mass of asymptotically hyperbolic Riemannian manifolds. Pacific Jour. Math **212**, 231–264 (2003). arXiv:math/0110035 math.DG

[10] Chruściel, P.T., Simon, W.: Towards the classification of static vacuum spacetimes with negative cosmological constant. Jour. Math. Phys. **42**, 1779–1817 (2001). arXiv:gr-qc/0004032

[11] Chruściel, P.T., Wutte, R.: Gluing-at-infinity of two-dimensional asymptotically locally hyperbolic manifolds. Class. Quant. Grav. **42**(24), 245007 (2025) arXiv:2401.04048 [gr-qc]

[12] Chruściel, P.T., Wutte, R.: Positivity of holographic energy. Phys. Rev. Lett. **136**(18), 181401 (2026). arXiv:2604.08183 [gr-qc] 10.1103/6bt4-r4q1 42172421

[13] Compere, G., Marolf, D.: Setting the boundary free in AdS/CFT. Class. Quant. Grav. **25**, 195014 (2008). arXiv:0805.1902 [hep-th]

[14] de Haro, S., Solodukhin, S.N., Skenderis, K.: Holographic reconstruction of space-time and renormalization in the AdS / CFT correspondence. Commun. Math. Phys. **217**, 595–622 (2001). arXiv:hep-th/0002230

[15] Fefferman, C., Graham, C.R.: The ambient metric. Ann. Math. Stud. **178**, 1–128 (2011). arXiv:0710.0919 math.DG

[16] Friedrich, H.: Einstein equations and conformal structure: Existence of anti-de-Sitter-type spacetimes. Jour. Geom. and Phys. **17**, 125–184 (1995)

[17] Henningson, M., Skenderis, K.: The Holographic Weyl anomaly. JHEP **07**, 023 (1998). arXiv:hep-th/9806087

[18] Hirsch, S., Zhang, Y.: Causal character of imaginary Killing spinors and spinorial slicings, (2025). arXiv:2512.14569 [gr-qc]

[19] Kamiński, W.: Well-posedness of the ambient metric equations and stability of even dimensional asymptotically de Sitter spacetimes. Commun. Math. Phys. **401**, 2959–2998 (2021). arXiv:2108.08085 [gr-qc]

[20] Kijowski, J., Tulczyjew, W.M.: A symplectic framework for field theories. Lecture Notes in Physics, vol. 107. Springer, New York, Heidelberg, Berlin (1979)

[21] Klinger, P.: Non-degeneracy of Riemannian Schwarzschild- Anti de Sitter metrics: Birkhoff-type results in linearized gravity, (2018). arXiv:1806.05023 [gr-qc]

[22] Lee, J., Wald, R.M.: Local symmetries and constraints. J. Math. Phys. **31**, 725–743 (1990)

[23] Papadimitriou, I., Skenderis, K.: Thermodynamics of asymptotically locally AdS spacetimes. JHEP **08**, 004 (2005). arXiv:hep-th/0505190

[24] Papadimitriou, I., Skenderis, K.: AdS / CFT correspondence and geometry. IRMA Lect. Math. Theor. Phys. **8**, 73–101 (2005). arXiv:hep-th/0404176

[25] Skenderis, K.: Asymptotically Anti-de Sitter space-times and their stress energy tensor. Int. J. Mod. Phys. A **16**, 740–749 (2001). arXiv:hep-th/0010138

[26] Wald, R.M., Zoupas, A.: A General definition of ‘conserved quantities’ in general relativity and other theories of gravity. Phys. Rev. D **61**, 084027 (2000). arXiv:gr-qc/9911095

[27] Wang, X.: The mass of asymptotically hyperbolic manifolds. Jour. Diff. Geom. **57**, 273–299 (2001)

